# Supplementary material for: Language bias in orthodontic systematic reviews: A meta-epidemiological study
Source: PLoS One. 2024 Apr 1;19(4):e0300881. doi: 10.1371/journal.pone.0300881 (PMC10984547; doi:10.1371/journal.pone.0300881)
Supplement: S1 Table — (DOCX) [file pone.0300881.s001.docx]

S1 Table : studies excluded with the reason.

| Study ID | Reason for excluding |
| --- | --- |
| Are orthodontic randomised controlled trials justified with a citation of an appropriate systematic review? | Methodological study |
| Artificial intelligence in orthodontics: Where are we now? A scoping review | scoping review |
| A tool for assessment of risk of bias in studies of adverse effects of orthodontic treatment applied in a systematic review on external root resorption | Methodological study |
| Development of a clinical practice guideline for orthodontically induced external apical root resorption | Guidelines |
| Corrigendum to: Effectiveness of self-applied topical fluorides against enamel white spot lesions from multi-bracketed fixed orthodontic treatment: a systematic review | Corrigendum |
| Intention-to-treat analysis: Are we managing dropouts and missing data properly in research on orthodontic treatment? A systematic review | Methodological study |
| Scoping review of systematic review abstracts about temporomandibular disorders: Comparison of search years 2004 and 2017 | Scoping review |
| Identification and appraisal of outcome measures used to evaluate hypodontia care: A systematic review | Methodological study |
| Extraction of primary (baby) teeth for unerupted palatally displaced permanent canine teeth in children | Withdrawn |
| Treatments for adults with prominent lower front teeth | Withdrawn |
| Orthodontic treatment for deep bite and retroclined upper front teeth in children | No included studies |
