# Supplementary material for: Language bias in orthodontic systematic reviews: A meta-epidemiological study
Source: PLoS One. 2024 Apr 1;19(4):e0300881. doi: 10.1371/journal.pone.0300881 (PMC10984547; doi:10.1371/journal.pone.0300881)
Supplement: S1 File — (DOCX) [file pone.0300881.s002.docx]

| **S1 File:** **Technical notes** |
| --- |

**Materials and methods**

***Addressing studies with unit-of-analysis issues***

Five out of 14 meta-analyses on the selected primary outcome suffered from unit-of analysis issues. These meta-analyses included at least one study comparing more than two interventions. Some meta-analyses already addressed the unit-of-analysis issue by splitting the sample size of the control arm as many times as the number of the remaining arms. For instance, a three-arm study comparing A versus B versus C (the control group) would appear twice in the dataset: A versus C and B versus C, after dividing the sample size of C approximately evenly in the two comparisons. Likewise, a four-arm study comparing A versus B versus C versus D (the control group) would appear thrice in the dataset: A versus D, B versus D, and C versus D after dividing the sample size of D approximately evenly in the three comparisons. This strategy *partially* addresses the unit-of-analysis issue and is described in the Cochrane Handbook as an option in this direction (1).

When performing a meta-analysis, we pursued this strategy in two meta-analyses that ignored the unit-of-analysis issue in the corresponding studies. Specifically, the systematic review of Fang and colleagues (2) included the study of Eissa and colleagues, who randomised 33 patients to receive Smart Track®, Damon, or regular brackets (3). The tooth length was measured before (B) and after (A) randomisation for four teeth (lateral and central on both sides). The authors reported the average change in tooth length (as a mean difference, MD) and standard deviation in each intervention and control group (left lateral, right lateral, left central, right central). We calculated the within-group correlation coefficient for each intervention and group using the formula in Cochrane Handbook (4),

$$r_{kl}=\frac{s_{kl,B}^{2}+s_{kl,A}^{2}-s_{kl,C}^{2}}{2\times s_{kl,B}\times s_{kl,A}}$$

with $r_{kl}$ being the correlation coefficient in intervention arm $k$ ($k=\text{Smart Track®, Damon, regular brackets}$) and group $l$ ($l=\text{left}\text{ }\text{lateral, }\text{righ}\text{t}\text{ }\text{lateral, }\text{left}\text{ }\text{central}\text{, }\text{right}\text{ }\text{central}$), $s_{kl,B}^{2}$ and $s_{kl,A}^{2}$ being the variance of tooth length in the intervention arm $k$ and group $l$ before (B) and after (A) randomisation, respectively, and $s_{kl,C}^{2}$ being the variance of the average change in the tooth length in the intervention arm $k$ and group $l$. We averaged the correlation coefficients and MDs across the four groups for each intervention,

$$\bar{r}_{k}=\frac{\sum_{l=1}^{4} r_{kl}}{4} \text{and} \bar{MD}_{k}=\frac{\sum_{l=1}^{4} {MD}_{kl}}{4}$$

Then, we calculated the variance of $\bar{MD}_{k}$ using the following formula,

$$S_{k,MD}^{2,Total}=Var\left( \bar{MD}_{k} \right)=\frac{1}{16}\left( \sum_{l=1}^{4} s_{kl,C}^{2}+2\times\bar{r}_{k}\times\sum_{i\neq j} s_{ki,C}\times s_{kj,C} \right)$$

where $\bar{MD}_{k}$ and $\bar{r}_{k}$ are the average MD in tooth length and the average correlation coefficient in the intervention arm $k$, respectively. Finally, we entered this study in the meta-analysis as two separate studies (Smart Track® versus regular brackets and Damon versus regular brackets) using the corresponding $\bar{MD}_{k}$ and $S_{k,MD}^{2,Total}$ and splitting the sample size of regular brackets into six and five patients for Smart Track® versus regular brackets and Damon versus regular brackets, respectively. Fang and colleagues (2) also included the multi-arm study of Wang and colleagues (5) with a design similar to Eissa and colleagues (3). We followed the same approach as Wang and colleagues (5) to obtain $\bar{MD}_{k}$ and $S_{k,MD}^{2,Total}$ for each intervention arm. Then, we entered this multi-arm study as separate studies for each intervention sharing the same control arm.

***Transforming from mean difference to standardised mean difference***

We preferred extracting arm-level data to contrast-level data when the former were available in the forest plot or elsewhere for the selected meta-analysis on the primary outcome. Arm-level data corresponds to the mean, standard deviation and sample size for each arm of every study for a continuous outcome. Contrast-level data refer to the estimated effect size and variance for each arm of every study. Of the 14 meta-analyses on the selected primary outcome, nine allowed us to extract arm-level data and five provided only contrast-level data.

We considered the standardised mean difference (SMD) as the effect measure for all extracted meta-analyses to make the results comparable within and across meta-analyses. Of the five meta-analyses on contrast-level data, one used SMD, and four used the mean difference (MD) as the effect measure. We used the formula of the variance of MD to obtain the pooled standard deviation for each study and transform the MD into SMD (6). The following formulas provided the pooled variance for a study with an unpaired or paired design, respectively,

$$S_{p}^{2}=\frac{V_{MD}{\times n}_{1}{\times n}_{2}}{n_{1}+n_{2}}$$

and

$$S_{p}^{2}=\frac{V_{MD}\times n}{2\times\left( r-1 \right)}$$

where $S_{p}^{2}$ is the pooled variance, $V_{MD}$ is the reported variance of MD, $n_{k}$ is the sample size of arm $k$ ($k=1,2$) in studies with unpaired design, $n$ is the sample size in studies with paired design, and $r$ is the correlation coefficient. We assumed equal variances in the compared arms for the studies with an unpaired design. We also assumed $r$ equal to 0.5 for the studies with a paired design. We used the Hedges’g to obtain an unbiased estimate of SMD (and variance thereof), with the bias being attributed to small samples (6). The correction factor for a study with an unpaired or paired design has the corresponding formula,

$$J=1-\frac{3}{4\times\left( n_{1}+n_{2}-2 \right)-1}$$

and

$$J=1-\frac{3}{4\times\left( n-1 \right)-1}.$$

We ensured the outcome had the same direction in all meta-analyses; a negative SMD favoured the intervention (first arm). Meta-analyses reporting positive SMD as favouring the intervention arm underwent a modification of their data to switch the direction. Specifically, we multiplied the mean by minus one in each arm of every study with arm-level data. We multiplied the SMD by minus one in each study with contrast-level data. Maintaining a consistent direction of the outcome across all meta-analyses allowed proper interpretation of the difference in SMD (ΔSMD) between non-English and English studies. Namely, a meta-analysis with a negative ΔSMD indicated that non-Engish studies tended to overestimate the treatment effect; whilst a positive ΔSMD indicated that Engish studies tended to overestimate the treatment effect. Furthermore, a consistent direction of the outcome allowed us to pool the ΔSMDs across the meta-analyses using a random-effects meta-analysis to obtain a summary ΔSMD and uncertainty thereof (see details below).

***Details on the statistical methods***

To investigate the association between statistical significance in the summary effect estimate and the inclusion of non-English studies, we restricted the sample to 29 SRs that conducted a meta-analysis and reported the language of the synthesised studies. We preferred a univariate binary logistic regression to the unconditional maximum likelihood estimator of OR due to the small sample of SRs, making it difficult to defend the latter. We reported the OR and 95% CI.

A two-stage approach was performed to analyse the meta-epidemiological data and estimate the average bias attributed to non-English studies. The meta-epidemiological data comprised meta-analyses on the primary outcome (one from each eligible SR) that included at least three studies, with at least one being non-English. The standardised mean difference (SMD) was the effect measure since all selected meta-analyses referred to a continuous primary outcome. Initially, a random-effects meta-regression with language type (non-English versus English) as the covariate was conducted in each meta-analysis. The intercept referred to the summary SMD in English studies, and the slope measured the summary difference in SMD (ΔSMD) between non-English and English studies. This model allowed the between-study variance to be estimated separately for each subgroup with at least two studies using the restricted maximum likelihood estimator for heterogeneity. Then, a random-effects meta-analysis was performed to combine the ΔSMDs across the meta-analyses. A negative ΔSMD would indicate that non-English studies overestimated SMD.

**Results**

***Examining the influence of non-English studies on summary results***

The meta-analyses included a median of 5 studies, ranging from 3 to 19 (interquartile range (IQR): 4 to 8). The total size of the included studies ranged from 12 to 1280, with a median of 32 patients (IQR: 24 to 47). Ten meta-analyses included only one non-English study. The remaining meta-analyses included a median of 4 non-English studies, ranging from 2 to 10 (IQR: 3 to 6). The included English studies ranged from 2 to 15 across all meta-analyses (median: 4, IQR: 3 to 7). English and non-English studies had quite similar sample sizes ranging from 12 to 113 (median: 30, IQR: 24 to 47) and 15 to 112 (median: 38, IQR: 26 to 47) patients, respectively. One English trial included a total of 1280 patients.

**References**

1. Higgins JPT, Eldridge S, Li T (editors). Chapter 23: Including variants on randomized trials. In: Higgins JPT, Thomas J, Chandler J, Cumpston M, Li T, Page MJ, Welch VA (editors). Cochrane Handbook for Systematic Reviews of Interventions version 6.3 (updated February 2022). Cochrane, 2022. Available from [www.training.cochrane.org/handbook](http://www.training.cochrane.org/handbook).
2. Fang X, Qi R, Liu C. Root resorption in orthodontic treatment with clear aligners: A systematic review and meta-analysis. Orthod Craniofac Res. 2019 Nov;22(4):259-269.
3. Eissa O, Carlyle T, El‐Bialy T. Evaluation of root length following treatment with clear aligners and two different fixed orthodontic appliances. A pilot study. J Orthod Sci. 2018;7:11. Eissa,
4. Higgins JPT, Li T, Deeks JJ (editors). Chapter 6: Choosing effect measures and computing estimates of effect. In: Higgins JPT, Thomas J, Chandler J, Cumpston M, Li T, Page MJ, Welch VA (editors). Cochrane Handbook for Systematic Reviews of Interventions version 6.3 (updated February 2022). Cochrane, 2022. Available from [www.training.cochrane.org/handbook](http://www.training.cochrane.org/handbook).
5. Wang G, Yang L, Zhang YF, Luo SL, Zheng JW. [A retrospective study on incisor root resorption in patients treated with bracketless invisible appliance and straight wire appliance]. Shanghai Kou Qiang Yi Xue. 2017;26(1):121‐124.
6. Borenstein M, Hedges LV, Higgins JPT, Rothstein HR. Effect sizes based in means. In: Introduction to Meta-analysis. John Wiley & Sons, Ltd; 2009.
