# Supplementary material for: Language bias in orthodontic systematic reviews: A meta-epidemiological study
Source: PLoS One. 2024 Apr 1;19(4):e0300881. doi: 10.1371/journal.pone.0300881 (PMC10984547; doi:10.1371/journal.pone.0300881)
Supplement: S1 Fig — (DOCX) [file pone.0300881.s003.docx]

| **S1 Figures** |
| --- |


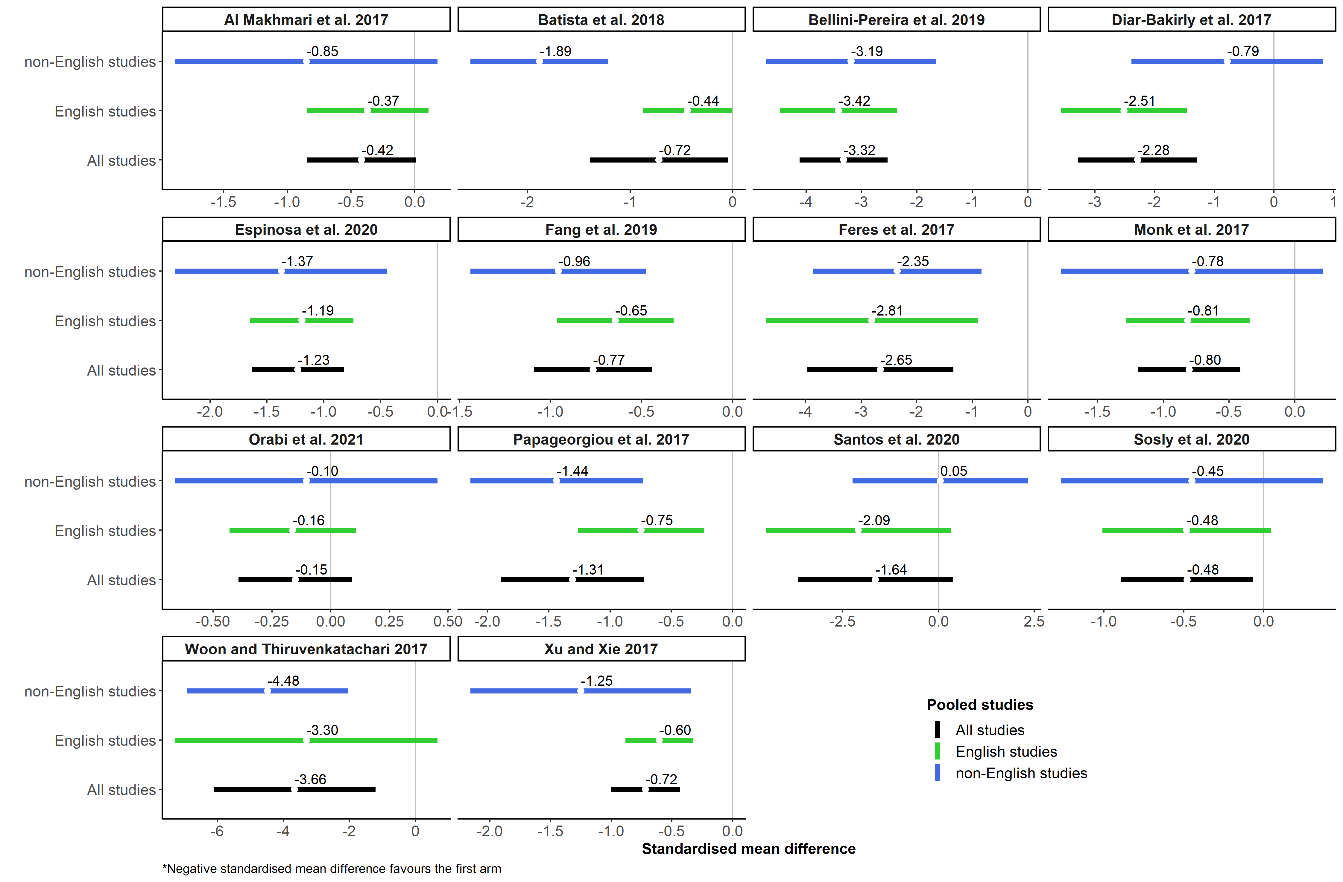


**Figure S1.** Forest plots on the summary standardised mean difference and 95% confidence interval in 14 meta-analyses on the selected primary outcome. Each panel present the meta-analysis results when all studies are pooled (black line), only English studies are pooled (green line), and only non-English studies are pooled (blue line). Confidence intervals that cross the vertical line at zero indicate statistically inconclusive evidence for the favoured arm (equivalent to p-value > 0.05).
