# Supplementary material for: Language bias in orthodontic systematic reviews: A meta-epidemiological study
Source: PLoS One. 2024 Apr 1;19(4):e0300881. doi: 10.1371/journal.pone.0300881 (PMC10984547; doi:10.1371/journal.pone.0300881)
Supplement: S2 Fig — (DOCX) [file pone.0300881.s004.docx]

| **S2 Figures** |
| --- |


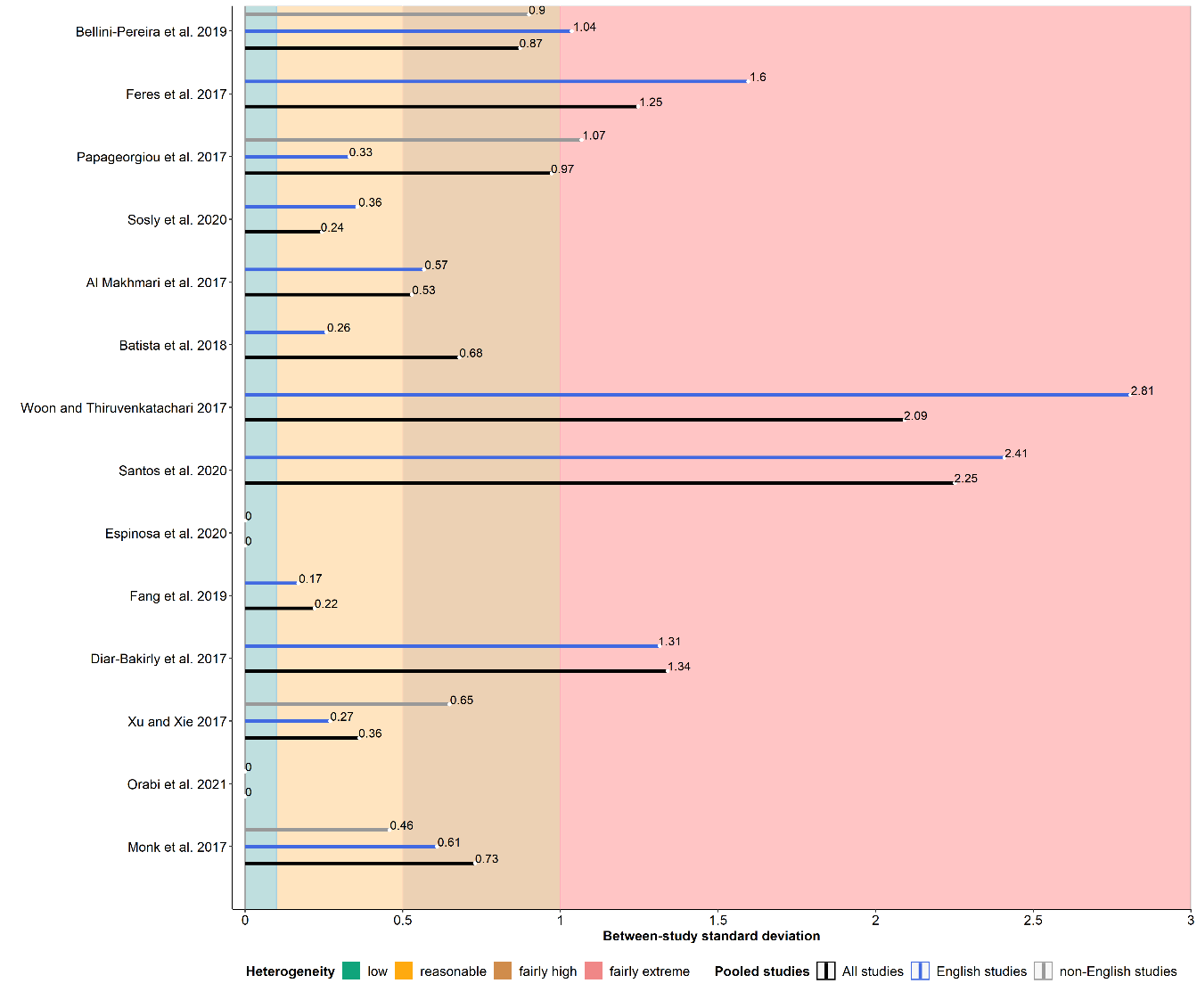


**S2 Figure.** Line plot on the between-study standard deviation (x-axis) in 14 meta-analyses (y-axis) on the selected primary outcome. The width of each horizontal line corresponds to the size of the estimated between-study standard deviation using the restricted maximum likelihood estimator. The different levels of statistical heterogeneity appear as green, yellow, orange, and red rectangles to indicate a low, reasonable, fairly high, and fairly extreme heterogeneity, respectively, following the classification of Spiegelhalter and colleagues (1). The between-study standard deviation is irrelevant for meta-analyses that included only one non-English study; hence, the corresponding grey horizontal line is missing.

References

Spiegelhalter DJ, Abrams KR, Myles JP. Bayesian approaches to clinical trials and health-care evaluation. John Wiley and Sons, Chichester, 2004.
