# Supplementary material for: Language bias in orthodontic systematic reviews: A meta-epidemiological study
Source: PLoS One. 2024 Apr 1;19(4):e0300881. doi: 10.1371/journal.pone.0300881 (PMC10984547; doi:10.1371/journal.pone.0300881)
Supplement: Attachment — Submitted filename: Revision letter R2.docx [file pone.0300881.s006.docx]

**Dear editor Luiz Alexandre Chisini,**

We would like to thank you and the reviewers for the comments and suggestions that we think will improve our manuscript.

Please find below responses and actions taken. In the revised manuscript we highlight amended sections.

**Additional Editor Comments:**

I have still concerns about the paper. The paper's objective is very interesting. However, the interpretation of the results is still incorrect. The authors remain doing affirmations that cannot be maintained by the statistics. So, the paper spin is very high and can lead the authors to misinterpretation.

- When the authors mention “174 SRs were eligible for inclusion in this study” it is not possible to undertint if all 174 were included or not for the revision. They are eligible for inclusion, but could not be included. So, it is better to readers understand the number of “included” instead of “eligible”. This is confusing in the abstract and in the results. Please, Make clear the number of articles included in the review and meta-analysis at the beginning of the results sections (in the abstract and results)

Authors’ response: the results and the abstract were amended according to the Editors’ comments, as follows:

Abstract (Results): ‘*174 SRs were included in this study*.’

Results (Systematic review selection): ‘*After full text reading of one hundred eighty-five SRs, 174 SRs were included in the present study*.’

- Please use the exact number of p-value.

Authors’ response: We have now added the p-value for the random-effects and fixed-effect model in the Results subsection ‘Examining the influence of non-English studies on summary results’.

- I have also concerns about the sentence: “As such, the evidence of the overestimation of meta-analysis results with non-English studies is inconclusive”. The meta-analysis with random models (which is the right model for studies with methodological variation) shows no difference. So, could be “inconclusive” only isn’t power enough, but then there are problems with the sample size. On the other hand, I believe that the conclusion is there was no evidence of significant overestimation.

Authors’ response: We amended the indicated sentence as follows: ‘*As such, the overestimation of meta-analysis results by including non-English studies was statistically non-significant*’.

- I dindnt found the result for this sentence: “The univariate binary logistic regression indicated that the odds of statistical significance in the summary effect estimate increased by 220% (OR: 3.20) in systematic reviews with non-English studies than in systematic reviews with only English studies. However, the evidence was weak because the 95% CI (0.57, 18.92) was substantially wide and included the null value”. Also, the authors cannot affirm that increase, because the 95% CI doesn’t allow this. Please, revise affirmations that cannot be supported by statistics.

Authors’ response: To avoid confusing the readers, we amended the indicated sentence as follows:

‘The univariate binary logistic regression indicated that the odds of statistical significance in the summary effect estimate was 3.20 times larger in systematic reviews with non-English studies than in systematic reviews with only English studies. However, the association was statistically non-significant (OR:3.20, 95%CI: 0.57, 18.92, P=0.18).’

- Here, another misinformation: “The present study found a positive, though, statistically non-significant association between including non-English studies in orthodontic SRs and SRs with small coauthorship or SRs led by an author affiliated in American institute”. The authors cannot affirm that the is a “positive” but non-significant association. Or there is an association or there is not. Please, revise.

Authors’ response: We removed ‘positive, though,’ from the indicated sentence.

- In the sentence “Our meta-epidemiological analysis demonstrated a larger summary effect size from including non-English studies, though the evidence was inconclusive due to material statistical heterogeneity”. Here again, they are not “inconclusive”.

Authors’ response: We replaced ‘*the evidence was inconclusive*’ with ‘*this was not statistically significant*’.

- This sentence “A wider search is unlikely to change our conclusions on the importance of including non-English studies.” Is a supposition and cannot be maintained with data. So, I recommend the authors to avoid this type of affirmation.

Authors’ response: We amended the indicated sentence as follows:

‘*A wider search may have some impact on the importance of including non-English studies.’*

- Both in the conclusion as the abstract conclusion, the authors cannot support this affirmation “Language restriction seems to have a non-negligible impact on the results of orthodontic SRs.” There was no change in the results according to the results. This interpretation leads to a spin on the study.
Authors’ response: Professors Doug Altman and Martin Bland wrote a seminar commentary to raise awareness on how results from statistical tests should be interpreted. The article’s title is ‘Absence of evidence is not evidence of absence’ (1); namely, finding a p-value above the selected level of significance (e.g., 5%), and thus, failing to reject the null hypothesis does not mean that there is no difference in the compared groups. It just means that the difference is statistically non-significant, and hence, we cannot draw any firm conclusion in favour or against any of the compared groups due to lack of sufficient information (e.g., small sample size) or substantial variation in the measurements, or a combination of both. Taken from the abstract of Altman and Bland: “*When statistical analysis of the study data finds a P value greater than 5%, it is convention to deem the assessed difference nonsignificant. Just because convention dictates that such study findings be termed nonsignificant, or negative, however, it does not necessarily follow that the study found nothing of clinical importance*.” (1). And lastly, from the last paragraph of the article: “*When we are told that “there is no evidence that A causes B” we should first ask whether absence of evidence means simply that there is no information at all. If there are data we should look for quantification of the association rather than just a P value. Where risks are small P values may well mislead: confidence intervals are likely to be wide, indicating considerable uncertainty.* ***While we can never prove the absence of a relation****, when necessary we should seek evidence against the link between A and B—for example, from case-control studies*.” (1)

We amended the indicated sentence as follows: ‘*Language restriction seems to have no statistically significant impact on the results of orthodontic SRs*’

**Reference**

(1) Altman DG, Bland JM. Absence of evidence is not evidence of absence. BMJ. 1995 Aug 19;311(7003):485. doi: 10.1136/bmj.311.7003.485.

In Table 2, please, standardize the reference category as the first row
In table 3, please, provide the p-value
Authors’ response: Tables were amended accordingly.

Reviewers' comments:

Reviewer's Responses to Questions

**Comments to the Author**

Reviewer #1: I would like to thank you for the opportunity to review this article again, as well as for the authors' thoughtful response. The authors have made considerable improvements to the paper that have enhanced the way the findings are communicated. Below are some minor corrections that can be made to ensure the best version of this manuscript.

Authors response: We would like to thank the reviewer for the valuable comments that have improved our manuscript. In the revised version, we are making efforts to incorporate additional clarification.

Abstract:
In the "Eligibility Criteria" section, it is clearly stated that the two main criteria for selecting SRs for inclusion were (1) publication between 2017 and 2021 and (2) being published in one of the five highest-impact journals indexed in PubMed, in addition to Cochrane reviews from the same period. In this way, I believe that in the "Data Source" section of the Abstract, the wording could be improved by inserting separately that the SRs were retrieved from five high-impact journals indexed in PubMed. After this, could be inserted that searches were carried out in the Cochrane database, also considering the publication period (2017-2021).

Authors’ response: We have amended the Data source section in Abstract as follows:

‘*SRs published in high-impact orthodontic journals between 2017 and 2021 were retrieved through an electronic search of PubMed. Additionally, Cochrane oral health group was searched in June 2022 for orthodontic systematic reviews published in the same period*.’

Methods:
In the response letter (page 8), the authors stated that in the Statistical Analysis and Data Synthesis subsection, two sentences had been included to address the issue of statistical significance interpretation in different analyses carried out. However, the second of these, copied below, was not present in the main text. Please revise the insertion of the information.
“•For the ΔSMD results: ‘We concluded a statistically significant ΔSMD when the 95% CI did not include the value 0, which coincides with a p-value below 5% (the significance level); otherwise, the result was statistically non-significant, which coincides with a p-value at least 5%”
Authors’ response: Thank you for this observation. We have now added all P values to the text for better clarity. However, if the reviewer thinks that we should add this sentence, we will be happy to follow his/her guidance.

Results:
Please verify Table S1: Although the Systematic review selection subsection mentions 11 excluded studies with reasons, only 10 titles are shown in Table S1.

Authors’ response: Sorry for this mistake. This excluded study was added to the table.

Reviewer #2: Most of my suggestions were accepted. The ones that couldn't be altered were justified accordingly. I appreciate the opportunity to review this study and recommend it for publication. Additionally, I would like to note that the authors have introduced an interesting and underexplored topic in the scientific literature.

Authors response: We would like to thank the reviewer for the valuable comments that have improved our manuscript.
